# Supplementary material for: Transcriptomic Insights into Mechanisms of Early Seed Maturation in the Garden Pea (Pisum sativum L.)
Source: Cells. 2020 Mar 23;9(3):779. doi: 10.3390/cells9030779 (PMC7140803; doi:10.3390/cells9030779)

Sprint−2(20) vs Zhongw

Global Summary

%DE = 0.87

genes with fdr < 0.2 = 540 (272 + / 268 −)

genes with fdr < 0.1 = 482 (237 + / 245 −)

genes with fdr < 0.05 = 403 (186 + / 217 −)

genes with fdr < 0.01 = 268 (113 + / 155 −)

genes in genesets = 0

<FC> = 0

<t−score> = −1.23

<p−value> = 0.01

<fdr> = 0.13

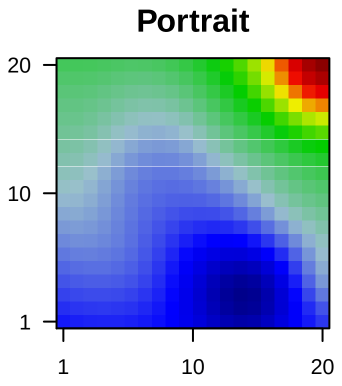

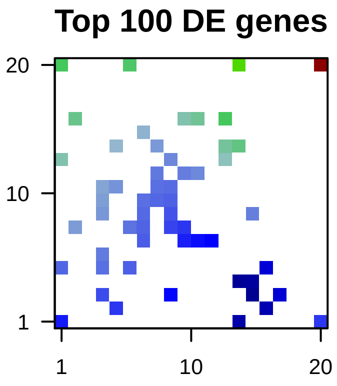

Global Genelist

Rank	ID	log(FC)	p−value / fdr / Metagene	Description
1	TRINITY_DN7	0	0.0000000100000009 x 11	
2	comp39525_c	0.26	0.0000000100000016 x 8	
3	TRINITY_DN1	0	0.0000000400000018 x 11	
4	TRINITY_DN4	0	0.0000000600000031 x 11	
5	comp22899_c	0.09	0.0000000100000031 x 5	
6	TRINITY_DN3	0	0.0000001300000035 x 11	
7	TRINITY_DN4	0	0.0000001700000059 x 11	
8	TRINITY_DN4	0	0.0000002600000059 x 11	
9	TRINITY_DN5	0	0.0000003800000059 x 11	
10	TRINITY_DN2	0	0.0000004000000058 x 10	
11	TRINITY_DN6	0	0.0000004600000058 x 11	
12	TRINITY_DN6	0	0.0000005600000058 x 10	
13	comp18340_c	−0.02	0.00000058000005010 x 12	
14	TRINITY_DN1	0	0.0000006000000057 x 10	
15	TRINITY_DN6	0	0.0000006700000138 x 10	
16	TRINITY_DN1	0	0.0000008300000139 x 11	
17	comp4201_c0	−0.03	0.0000010000000212 x 8	
18	TRINITY_DN1	0	0.0000012600000264 x 6	
19	TRINITY_DN3	0	0.0000021400000269 x 11	
20	TRINITY_DN3	0	0.0000023900000269 x 11	

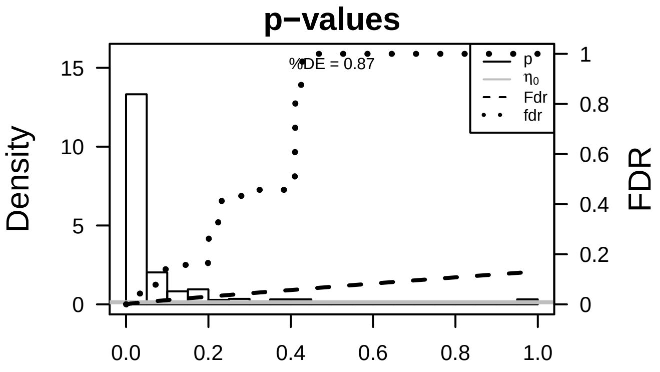

Supplement: Supplementary file 1 [file cells-09-00779-s001.zip › Supplementary materials/FileS3.zip!/FileS2/Hormones - Results/Summary Sheets - Differences/Reports/Sprint.2.20..vs.Zhongwan.6.10..pdf]
